# Itch and skin rash from chocolate during fluoxetine and sertraline treatment: Case report

**DOI:** 10.1186/1471-244X-4-36

**Published:** 2004-11-02

**Authors:** Jonas Cederberg, Stefan Knight, Svante Svenson, Håkan Melhus

**Affiliations:** 1 Department of Clinical Chemistry and Pharmacology, Uppsala Academic Hospital, Uppsala, Sweden; 2 Department of Molecular Biology, Swedish University of Agricultural Sciences, Uppsala, Sweden; 3 Fålhagens Primary Care Center, Uppsala, Sweden

## Abstract

**Background:**

The skin contains a system for producing serotonin as well as serotonin receptors. Serotonin can also cause pruritus when injected into the skin. SSRI-drugs increase serotonin concentrations and are known to have pruritus and other dermal side effects.

**Case presentation:**

A 46-year-old man consulted his doctor due to symptoms of depression. He did not suffer from any allergy but drinking red wine caused vasomotor rhinitis. Antidepressive treatment with fluoxetine 20 mg daily was initiated which was successful. After three weeks of treatment an itching rash appeared. An adverse drug reaction (ADR) induced by fluoxetine was suspected and fluoxetine treatment was discontinued. The symptoms disappeared with clemastine and betametasone treatment. Since the depressive symptoms returned sertraline medication was initiated. After approximately two weeks of sertraline treatment he noted an intense itching sensation in his scalp after eating a piece of chocolate cake. The itch spread to the arms, abdomen and legs and the patient treated himself with clemastine and the itch disappeared. He now realised that he had eaten a chocolate cake before this episode and remembered that before the first episode he had had a chocolate mousse dessert. He had never had any reaction from eating chocolate before and therefore reported this observation to his doctor.

**Conclusions:**

This case report suggests that there may be individuals that are very sensitive to increases in serotonin concentrations. Dermal side reactions to SSRI-drugs in these patients may be due to high activity in the serotonergic system at the dermal and epidermo-dermal junctional area rather than a hypersensitivity to the drug molecule itself.

## Background

The skin contains a system for producing serotonin as well as serotonin receptors. Serotonin can also cause pruritus when injected into the skin. SSRI-drugs increase serotonin concentrations and are known to have pruritus and other dermal side effects *e.g. *exanthema, purpura, urticaria and pruritus [1]. In contrast, SSRI-medication has also been used to treat pruritus associated with cholestasis [2] and polycythemia vera [3]. In this report we describe a patient who developed pruritus and skin rash from chocolate, but only when he was under SSRI-treatment. The case is presented and we provide a putative biological rationale for the described phenomenon.

## Case presentation

A 46-year-old man consulted his doctor in September 2003 due to depression. He had then experienced symptoms for a few years that had aggravated during the last six to eight months. Using the Montgomery-Åsberg Depression Rate Scale (MADRS) the patient scored 24 points and was diagnosed as having a clinical depression. He did not take any medication and had no regular medical contact. The patient did not have any history of allergy or dermatological diseases. However, he sometimes suffered from vasomotor rhinitis after drinking red wine. The doctor prescribed fluoxetine 20 mg daily as antidepressive treatment. At the revisit three weeks later the patient was very pleased with the fluoxetine treatment and reported that he "had not felt better in 20 years" although he initially had experienced slight nausea and insomnia.

A week later, he visited his doctor due to an itching rash that had started the day before. The doctor noted partly confluent urticae on the abdomen, a modest periorbital oedema and red, warm palms and wrists. An ADR induced by fluoxetine was suspected and fluoxetine treatment was discontinued. The symptoms were treated with 2 mg clemastine and 6 mg betametasone orally and disappeared within 48 hours. However, the symptoms of depression returned. Sertraline medication was initiated 10 days after the cessation of fluoxetine treatment since SSRI medication had shown good effect. During the weeks of sertraline treatment no urticarial symptoms appeared. The patient improved in his depression although full recovery was not achieved this time. After approximately two weeks of sertraline treatment he noted an intense itching sensation in his scalp after eating a piece of chocolate cake. The itch spread to the arms, abdomen and legs within a few hours. This time the patient did not seek his doctor but treated himself with clemastine and the itch disappeared during the night. He now remembered that he had had a chocolate mousse dessert before the first episode. Since he had never had any reaction from eating chocolate before, he found this observation so striking that he reported it to his doctor. The patient, himself a scientist, later tried small doses of chocolate and skin rash and itch appeared at an intensity that to him seemed dependent on the "dose" of chocolate ingested.

It has been known for 30 years that serotonin can stimulate cutaneous C-fibres [4], the type of fibres that is also known to transmit itch [5]. Moreover, serotonin injections into the skin can induce itch [6] and pruritus is a component in 24% of reported skin reactions to fluoxetine in Sweden, the corresponding figure for sertraline is 15 % [1]. However, attempts to treat pruritus using 5-HT3-receptor-antagonists have not given clear-cut results [6-8]. The enzymes necessary for conversion of tryptophan to serotonin are expressed in human skin [9]. In addition, 5-HT2AR are present in one third of unmyelinated axons at the dermal and epidermo-dermal junctional area [10]. An altered localisation pattern of serotonin receptors 5-HT1AR, 5-HT2AR and 5-HT3R has been reported in contact eczematous skin together with increased serotonin concentrations [11,12] indicating the presence of a serotonin system in the skin that can be altered in pathologic conditions. Moreover, a cross-sensitivity has been reported when skin rash developed after both paroxetine and sertraline medication [13]. Since these substances are structurally different, one interpretation is that the skin can react to an SSRI-induced increase in serotonin concentrations.

In the present case the patient experienced skin symptoms from two different SSRIs. However, these symptoms occurred only when he had eaten chocolate. Chocolate contains serotonin, at concentrations which depend on the type of chocolate [14]. A concentration of 1.4 – 5 μg / g has been reported in dark chocolate [14]. The present report suggests an interaction between SSRI-medication and chocolate leading to pruritus and rash. A plausible explanation is that SSRI together with serotonin-containing chocolate has increased serotonin concentration to a level where 5-HT receptors system at the dermal and epidermo-dermal junctional area are affected. Moreover, the patient in this case had previously noted nasal congestion and cough when he was drinking red wine. Red wine can induce release of serotonin from platelets [15] and from the gut [16]. Serotonin can induce nasal itch, sneeze and hypersecretion [17,18].

## Conclusions

Apart from the SSRI – chocolate interaction this patient had another possible sign of sensitivity to serotonin. The present case thus suggests that there may be individuals that are very sensitive to increases in serotonin concentrations. Skin side reactions to SSRI-drugs in these patients may be due to high activity in the serotonergic system system at the dermal and epidermo-dermal junctional area rather than a hypersensitivity to the drug molecule itself. However, the reaction of skin to serotonin from food is poorly studied and further studies are necessary to determine how much alimentary serotonin can increase serum serotonin concentrations and to what extent SSRI-medication affects this process. More knowledge in this field could be of help for physicians who encounter patients with dermal reactions to SSRI-drugs and there might be food and beverages containing serotonin that these patients should avoid. Moreover, possible individual differences in the serotonergic system at the dermal-epidermal junction remain to be studied.

What happened to the patient and his depression? Due to poor anti-depressive effect of sertraline, the treatment was altered back to fluoxetine. He is now free from his depression and experiences no rash or oedema-like adverse reactions as long as he is avoiding chocolate.

## List of abbreviations

5-HT: 5-hydroxytryptamine, ADR: Adverse Drug Reaction, SSRI: serotonin selective reuptake inhibitors

## Competing interests

The author(s) declare that they have no competing interests.

## Authors' contributions

SS first described the case, JC and HM performed literature searches and JC first drafted the manuscript. HM and SK took part in the scientific discussion and in finalising the manuscript.

## Pre-publication history

The pre-publication history for this paper can be accessed here:


